# Natural Killer Cells in SARS-CoV-2 Infection: Pathophysiology and Therapeutic Implications

**DOI:** 10.3389/fimmu.2022.888248

**Published:** 2022-06-30

**Authors:** Clara Di Vito, Francesca Calcaterra, Nicolò Coianiz, Sara Terzoli, Antonio Voza, Joanna Mikulak, Silvia Della Bella, Domenico Mavilio

**Affiliations:** ^1^Unit of Clinical and Experimental Immunology, IRCCS Humanitas Research Hospital, Milan, Italy; ^2^Department of Medical Biotechnologies and Translational Medicine (BioMeTra) , University of Milan, Milan, Italy; ^3^Department of Biomedical Sciences, Humanitas University, Milan, Italy; ^4^Emergency Medicine Unit, IRCCS Humanitas Research Hospital, Milan, Italy

**Keywords:** NK cells, COVID-19, SARS-CoV-2 infection, memory-like, immunotherapy, single cell sequencing

## Abstract

Natural Killer (NK) cells are lymphocytes of the innate immunity that play a crucial role in the control of viral infections in the absence of a prior antigen sensitization. Indeed, they display rapid effector functions against target cells with the capability of direct cell killing and antibody-dependent cell-mediated cytotoxicity. Furthermore, NK cells are endowed with immune-modulatory functions innate and adaptive immune responses *via* the secretion of chemokines/cytokines and by undertaking synergic crosstalks with other innate immune cells, including monocyte/macrophages, dendritic cells and neutrophils. Recently, the Coronavirus disease 2019 (COVID-19) pandemic, caused by severe acute respiratory syndrome coronavirus 2 (SARS-CoV-2), has spread globally. Although the specific role of NK cells in COVID-19 pathophysiology still need to be explored, mounting evidence indicates that NK cell tissue distribution and effector functions could be affected by SARS-CoV-2 infection and that a prompt NK cell response could determine a good clinical outcome in COVID-19 patients. In this review, we give a comprehensive overview of how SARS-CoV-2 infection interferes with NK cell antiviral effectiveness and their crosstalk with other innate immune cells. We also provide a detailed characterization of the specific NK cell subsets in relation to COVID-19 patient severity generated from publicly available single cell RNA sequencing datasets. Finally, we summarize the possible NK cell-based therapeutic approaches against SARS-CoV-2 infection and the ongoing clinical trials updated at the time of submission of this review. We will also discuss how a deep understanding of NK cell responses could open new possibilities for the treatment and prevention of SARS-CoV-2 infection.

## 1 Introduction

### 1.1 Natural Killer Cells: General Features

Natural Killer (NK) cells are innate lymphocytes that play a critical role in the primary immunological response to viral infections and in tumor surveillance. They display rapid effector functions with the capability of direct target cell killing and antibody-dependent cell-mediated cytotoxicity (ADCC) (1, 2). Furthermore, NK cells are endowed with immune-modulatory functions regulating and linking innate and adaptive immune responses *via* the secretion of chemokines/cytokines and by undertaking synergic crosstalks with antigen-presenting cells (APCs) (3).

Under homeostatic conditions, NK cells represent about 5-15% of circulating lymphocytes and are subdivided into two distinct subsets of CD56^bright^/CD16^neg^ (CD56^bright^) and CD56^dim^/CD16^pos^ (CD56^dim^) (4, 5). The CD56^bright^ cell subset accounts up to 10% of the whole blood NK cell population and mainly exerts important regulatory functions [i.e., production of soluble mediators such as interferon (IFN)-γ and tumor necrosis factor (TNF)-α, and establishment of cellular interplays]. Conversely, CD56^dim^ NK cells (up to 90% of the whole blood NK cell population) were primarily reported to act as cytotoxic effectors. Different subsets of human NK cells have been also described in peripheral tissues. The tissue-specific human NK cell populations often carry phenotypic hallmarks that distinguish them from their circulating counterparts and are present under homeostatic conditions in both secondary lymphoid organs (6, 7) and non-lymphoid organs, including decidua or liver (8, 9).

In addition to canonical NK cells, increasing evidence demonstrated the existence of tissue-resident and circulating NK cells endowed with adaptive-like features. These adaptive/memory-like NK cells have been firstly described in response to Cytomegalovirus (CMV) infection and re-activation and are characterized by more vigorous functional responses, longer life span and more resistance to immune suppression than the other NK cell subsets (10, 11).

NK cell activation and functions are regulated by the interplay between a large number of inhibitory and activating receptors in combination with the presence of certain cytokines (1, 12). Together, these stimuli determine the type and strength of NK cell activity in terms of cytokine secretion and killing of target cells. Major activating receptors are the natural cytotoxicity receptors (NCRs) NKp46, NKp30, and NKp44 that are Ig-like transmembrane proteins. NKp46 and NKp30 are expressed on virtually all resting NK cells, whereas NKp44 expression is acquired upon NK cell activation. These molecules are important for inducing NK cell cytotoxic function against target stressed-cells and in the crosstalk with other cell types, such as dendritic cells (DCs) (13). Other important activating NK receptors are the C-type lectin-like receptors NKG2D and NKG2C and the activating Killer Immunoglobulin-Like Receptors (KIRs). NK cells are also equipped with several activating co-receptors including DNAX accessory molecule (DNAM-1), NKp80, 2B4 and NTB-A, capable of amplifying the NK cell triggering induced by NCRs or NKG2D. In addition, NK cells are activated through binding to antibody-opsonized target cells with CD16, Fc-γ receptor IIIA, which induces ADCC. Of note, CD16 is the only receptor that can activate NK cells on its own, without any additional activation through other receptors (14). Moreover, NK cells may express toll-like receptors (TLRs) that, after interaction with bacterial or viral products and in the presence of pro-inflammatory cytokines, induce potent NK cell activation (15).

NK cells are able to recognize and spare self cells from the killing, thanks to the expression of major histocompatibility complex class I (MHC-I) molecules, which interact with inhibitory receptors present on the NK cell surface. This inhibitory receptor-mediated signaling is essential to counteract activating signaling in order to protect against NK cell over-activity. This mechanism of target cell recognition *via* the absence of inhibitory MHC-I engagement is known as the “missing-self” hypothesis (16). Human NK cells express two main classes of HLA-class I-specific inhibitory receptors: members of the KIR superfamily and the CD94/NKG2A heterodimer (12, 17). KIRs are type I transmembrane receptors specific for polymorphic HLA-A, -B and -C molecules, whereas NKG2A is a type II transmembrane receptor of the C-type lectin-like receptor family that recognizes HLA-E, a non-classical HLA molecule characterized by limited polymorphism. Importantly, KIRs are characterized by high levels of polymorphism, which may affect KIR/HLA interactions. In fact, certain KIR/HLA combinations have been shown to correlate with protection or susceptibility to several human disorders (18).

### 1.2 NK Cell-Mediated Antiviral Mechanisms

In humans, NK cells are important mediators of the responses against viruses, including members of the herpesvirus, retroviruses, poxvirus and papilloma virus families. In fact, patients with identified NK cell deficiencies are predisposed to particularly severe, recurrent viral infections (19).

NK cells have multiple mechanisms to kill virus-infected cells.

The most important one is represented by the ability of some viruses to downregulate surface expression of MHC class I on the host cell surface to interfere with the presentation of viral antigens to T cells (20). According to the ‘missing self’ hypothesis, this decreased MHC-I expression promotes the recognition and clearance of virus-infected target cells by NK cells.

Accumulating evidence has revealed the importance of NK cell-activating receptors in antiviral defense (21). For instance, NCRs are known to bind viral glycoproteins, allowing NK cell activation upon detection of infected cells. NKG2D binds ligands on virally infected cells, including MHC class I polypeptide-related sequence A (MICA), MICB and the RAET1/ULBP family of proteins. Also, NKG2C receptor is renowned as the receptor that recognizes polymorphic CMV peptides. Furthermore, NKp80 and co-activating receptors DNAM1 and CD2 increase antiviral NK response. In addition, NK cells express multiple extracellular ligands, including Fas ligand (FasL) and the tumor necrosis factor-related apoptosis-inducing ligand (TRAIL) which engagement mediate cytolysis of target cells (22). As is known, viruses such human CMV or encephalomyocarditis virus (EMCV) induce the expression of death receptors on infected cells, which can subsequently interact with FasL and TRAIL on NK cells, resulting in apoptosis of the target cell (23).

In addition to cytotoxicity, NK cells contribute to the antiviral response through the release of a wide range of proinflammatory cytokines with antiviral activity. In particular, INFs and IFN-induced cytokines program immune cells to mount responses that promote viral control (24, 25). Distinct genetic associations between KIRs expressed on NK cells and their specific HLA haplotypes also affect viral infections. For example, the presence of *KIR3DS1* combined with *HLA-Bw4-I80* allele in patients with human immunodeficiency (HIV) infection has a protective effect and is associated with lower viral load and delayed progression to Acquired Immunodeficiency Syndrome (AIDS) (26).

Finally, NK cells can eliminate virus-infected cells *via* CD16-mediated ADCC. In fact, NK cell-mediated ADCC prevents HIV infection *via* the engagement of Fcγ receptors after the administration of the anti-HIV neutralizing Ab (NAbs) (27).

Although NK cells are essential in the early response against viral infections, through the killing of virus-infected cells, several viruses have evolved multiple mechanisms to evade NK cell-mediated viral clearance that affect NK cell phenotype and effector functions.

### 1.3 NK Cells in Coronavirus Infections

Coronaviruses are a group of enveloped single-stranded RNA viruses having an extensive range of natural hosts, including a variety of economically important vertebrates and humans. Indeed, seven coronaviruses have been known to infect human hosts causing respiratory diseases. Among them, Severe Acute Respiratory Syndrome Coronavirus (SARS-CoV) and Middle East respiratory syndrome coronavirus (MERS-CoV) are zoonotic and highly pathogenic coronaviruses that have resulted in regional and global outbreaks in the last decades. In 2019, a third zoonotic coronavirus, named Severe Acute Respiratory Syndrome Coronavirus 2 (SARS-CoV-2), causing the Coronavirus disease 2019 (COVID-19) has spread globally (28).

The specific role of NK cells in Coronavirus disease pathophysiology still needs to be explored, however, several studies in both SARS-CoV-1 and SARS-CoV-2 suggest that NK cells could be affected by these infections (29, 30).

Seminal studies in SARS-CoV-1 infected subjects demonstrated that the number of circulating NK cells and the expression of the inhibitory KIR CD158b is reduced with respect to those in healthy individuals and patients affected by Mycoplasma pneumoniae infection. This correlated with disease severity and the presence of anti-SARS coronavirus-specific antibodies (31). Moreover, the reduction of circulating NK cells in SARS-CoV-1 infected subjects persisted for the first 4 weeks after the appearance of symptoms (32). In this context, in a murine model mimicking the human SARS-CoV-1 infection, it has been hypothesized that the reduction of circulating NK cells could be due to their migration to the lung in response to several chemokines and cytokines, including CXCL10, CCL2, CCL3, and CCL5, TNF-α and interleukin (IL)-6 (29).

In this review, we provide a comprehensive overview of how SARS-CoV-2 infection interferes with the antiviral effector functions of NK cells and with the interactions between NK cells and other innate immune cells. Moreover, we will summarize the current ongoing clinical trials aiming at “fine tuning” NK cell activity in the context of COVID-19.

## 2 NK Cells in SARS-CoV-2 Infection

### 2.1 NK Cell Redistribution During SARS-CoV-2 Infection

**Figure 1:**
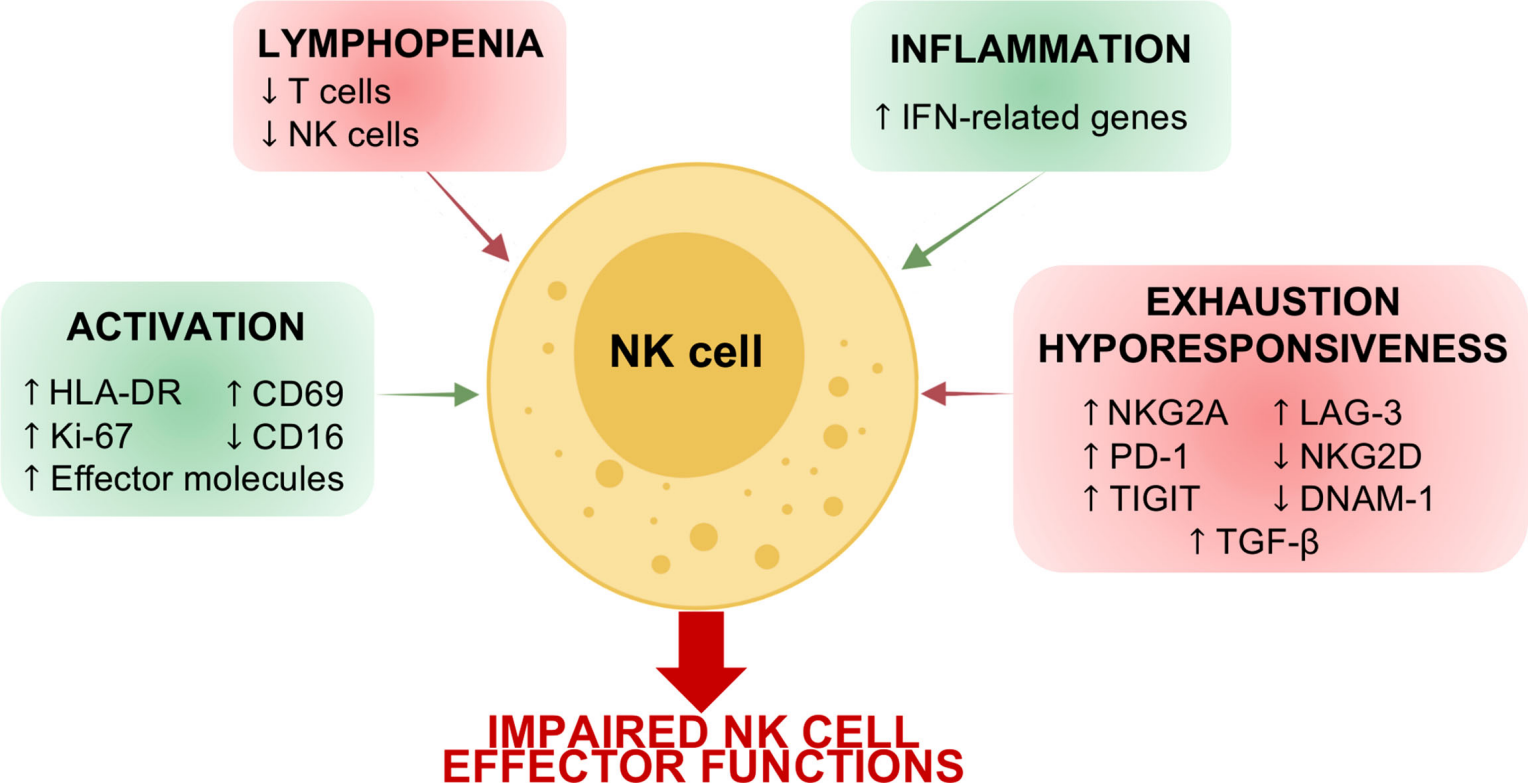
Schematic representation of COVID-19 effects on NK cells. Acute SARS-CoV-2 infection affects the number of circulating NK cells and their phenotype. Indeed, due to the local and systemic inflammation, NK cells in COVID-19 patients are characterized by a signature attributable to cell activation and inflammation as well as to cell exhaustion and hyporesponsiveness. These alterations in NK cell phenotype determine an impairment of NK cell effector functions in terms of IFN-γ and TNF-α production, degranulation cytolytic potential and ability to control virus replication.

Patients affected by SARS-CoV-2 infection are lymphopenic. This lymphopenia is often associated with neutrophilia and monocytopenia, especially in severely infected individuals (33). Specifically, the severe clinical presentation of COVID-19 is characterized by reduced T cell (CD4^pos^ Th1, Tregs, and CD8^pos^ T cells) counts with respect to non-infected subjects and to mild cases. Similarly, several independent reports indicated that the number of NK cells in the bloodstream is also affected by SARS-CoV-2 infection, without differences in NK cell subset distribution (**Figure 1**) (34, 35). This decrease in circulating NK cells seems to be directly correlated with the acute phase of the disease and with disease severity (36, 37). Indeed, it has been demonstrated that NK cell counts, as well as the T cell counts, are restored in late stages of the disease, while patients with a fatal course of the disease show a gradual loss of NK cells after the onset of symptoms (34, 35, 38). In agreement, recent findings demonstrated that NK cell counts in hospitalized patients is directly related to the speed of viral load decline. In particular, patients with “normal” (> 40 cell/μl) NK cell numbers show a faster decline of viral load compared to those with “low” (≤ 40 cell/μl) NK cell numbers, independently from the clinical status (39); thus, suggesting that circulating NK cell counts could represent a prognostic clinical parameter to predict the outcome of COVID-19.

Latest works also investigated the impact of SARS-CoV-2 infection in convalescent patients. Several studies observed a significant increase in circulating NK cells after the resolution of the infection (40, 41), while others showed normal NK cell counts in convalescent individuals (34, 42). These contrasting results could be due to the timing of analysis, to the COVID-19 severity of the selected patient cohort as well as to the presence of subjects with Long-COVID, a post-acute COVID syndrome with physical and neuropsychiatric symptoms lasting longer than 12 weeks after the resolution of the infection. Indeed, patients affected by Long-COVID have high levels of circulating NK cells with respect to recovered subjects (43).

Consistently with previous findings in SARS-CoV infection (29, 31, 32), it has been hypothesized that the depletion of circulating NK cells could be due to a redistribution of these lymphocytes from the blood and to their sequestration to the lung. Accordingly, single-cell RNA sequencing (scRNA-seq) analysis of bronchoalveolar lavage fluids (BALFs) from COVID-19 patients confirmed higher amounts of NK cells in the lung during the acute phase compared to controls; thus, suggesting that NK cells could potentially contribute to exacerbate lung tissue damage and epithelial cell death (44). In agreement with this hypothesis, serum analysis of SARS-CoV-2 positive subjects revealed a generalized inflammatory state with increased levels of several pro-inflammatory cytokines. Among them, the levels of CXCL16, involved in the migration of NK cells from the blood towards the infected airways, appears to be elevated early in the acute phase in both mild and severe SARS-CoV-2 infected patients (45, 46). Furthermore, the levels of CXCL10, a key chemokine produced by activated bronchial and alveolar epithelial cells in response to infections and involved in the etiology of various pulmonary conditions (such as pulmonary fibrosis), have been found to be increased early in COVID-19 patients. Like CXCL16, CXCL10 attracts NK cells, as well as Th1 and CD8^pos^ T cells, into the lungs *via* CXCR3 engagement and it is implicated in T cell apoptosis (46). Of note, it has been suggested that this chemokine in both the periphery and alveolar compartments could be involved in determining the clinical outcome of COVID-19 patients. Indeed, CXCL10 concentration is higher in COVID-19 deceased patients, and it is directly correlated with the duration of mechanical ventilation in subjects with acute respiratory distress syndrome (ARDS) due to SARS-CoV-2 infections (47, 48). In addition, BALF from COVID-19 patients contains elevated levels of other chemokines that potentially could attract NK cells, including CCL3, CCL3L1, CCL4, CXCL9, and CXCL11 (44). As a matter of fact, the characterization of NK cells within BALF and blood revealed the enrichment in transcripts for CXCR3, CXCR6, and CCR5 in the lung and the loss of these lung-homing potential markers within circulating NK cells in COVID-19 patients; thus further corroborating the hypothesis of the NK cell redistribution in the infected lung tissue (49).

### 2.2 Impact of SARS-CoV-2 Infection on NK Cell Phenotype

#### 2.2.1 NK Cell Receptor Expression

Like other viruses, including influenza virus, CMV, and HIV, SARS-CoV-2 can exhibit a variety of evasion strategies to interfere with NK cell functions and to overcome their antiviral cell responses, by modulating NK cell receptor expression, signaling and cytokine secretion.

In this regard, HLA-E is overexpressed in immune and stromal cells in BALF of COVID-19 patients and SARS-CoV-2 spike protein seems to be involved in this upregulation (36, 50).

Of note, several findings reported that the inhibitory receptor NKG2A is highly expressed by circulating NK cells in COVID-19 patients during the acute phase (**Figure 1**) (34, 36, 51). The expression of NKG2A also correlated with an NK cell inflammatory signature in patients with COVID-19, suggesting that NKG2A^pos^ NK cells could mediate anti-viral activity in the lung microenvironment (52). Furthermore, while mild and moderate patients show a recovery of basal levels of NKG2A expression on NK cells after the resolution of the infection, in severe convalescent subjects this inhibitory receptor is still upregulated (53).

On the other hand, other experimental evidence demonstrated that NKG2A is downregulated in COVID-19 patients and that this downregulation is counterbalanced by the upregulation of NKG2C, the activating counterpart of NKG2A, especially in severe ones. However, whether the timing of analysis, the COVID-19 severity as well as the presence of different SARS-CoV-2 variants are involved in determining these opposite results remains to be determined.

The experimental findings focusing on NKG2C^pos^ NK cells in SARS-CoV-2-infected individuals demonstrates that these cells are also characterized by a higher expression of CD57 and KIRs (36, 54, 55). Accordingly, Varchetta and coworkers have observed the expansion of CD57^pos^ FcϵRIγ^neg^ NK cells in COVID-19 patients with a poor outcome compared to survivors (54). Moreover, this signature identifies adaptive-like NK cells in humans and have been mainly characterized in CMV infection/reactivation (5, 56–59). In a first attempt, to disclose the possible contribution of CMV in driving the expansion of adaptive-like NK cells in SARS-CoV-2 infected subjects, Maucourant *et al.* observed that most of the severe COVID-19 patients analyzed had no detectable circulating CMV DNA, despite the expansion of NKG2C^pos^ NK cells was confined to seropositive individuals (36). These data thus suggest that the expansion of adaptive-like NK cells in severe patients is independent on CMV reactivation secondary to COVID-19. Despite these findings, is still to be determined whether adaptive-like NK cells accumulate in the blood during SARS-CoV-2 infection due to a higher resistance to cytokine-induced apoptosis (57) or if SARS-CoV-2 could drive the expansion of adaptive-like NK cells directly or indirectly through the hyper-production of pro-inflammatory cytokines (60, 61).

Furthermore, since the expansion of these NK cells showing an adaptive-like phenotype is related to a poor prognosis in COVID-19 patients (36, 54, 62), there is an urgent need to better understand the real ability of NK cell subpopulations to control SARS-CoV-2 infection and to mediate recall responses. In this regard, preliminary evidence suggests that NKG2C^pos^CD57^pos^ NK cells from convalescent subjects can mount a specific immune response against soluble SARS-CoV-2 peptides by secreting IFN-γ (53). On the contrary, very recent findings in Long-COVID patients demonstrated that while CD56^pos^CD57^pos^NKG2C^pos^ NK cell subpopulation is still expanded, their virus-specific and aspecific effector-functions are impaired (43).

It is plausible that the expansion of NKG2C^pos^ NK cells, founded especially in CMV-seropositive individuals and in aged patients, and the resulting contraction of NKG2A^pos^ NK cell pool, can lowering the HLA-E-restricted missing self-responses, potentially resulting in a reduced anti-SARS-CoV-2 immunity. Thus, the investigation of the balance between NKG2A^pos^ and NKG2C^pos^ NK cells could allow a better comprehension of patient-specific NK cell effector-functions and could represent a prognostic tool in COVID-19 patients both during the acute phase of the disease as well as after the resolution of the infection.

In addition to the above-mentioned NK cell receptors, the KIR haplotype is emerging as an important aspect in determining the disease severity. Indeed, the Bx genotype has been found more commonly associated to COVID-19 onset than AA genotype. However, patients harboring the Bx genotype have mainly a mild disease (63). In agreement, it has been shown that the expression of KIR2DS5 is associated to a shorter time to recovery, while the expression of KIR2DS2 has a protective role against SARS-CoV-2 infection (64, 65). On the contrary, patients harboring the KIR2DS4 and KIR2DL3 genes of the A haplotype exhibit the highest risk for severe COVID-19 (63).

By investigating the expression of NCRs in NK cells from SARS-CoV-2 infected subjects, several independent laboratories reported any changes in their frequencies compared to healthy individual. Only NKp44 was found slightly increased, especially in severe hospitalized COVID-19 patients (39, 54, 62).

#### 2.2.2 Inflamed and Activated Signature

In addition to the deregulation of the above-mentioned NK cell markers, a robust NK cell activation and proliferation was observed in peripheral blood and BALF from COVID-19 patients. Indeed, seminal studies demonstrated that COVID-19 patients show an upregulation of HLA-DR and CD69, together with the proliferation marker Ki-67 (**Figure 1**) (36). In agreement, very recent scRNA-seq data showed that in COVID-19 patients, particularly in severe ones, proliferating NK cells are expanded (39, 66).

**Figure 2:**
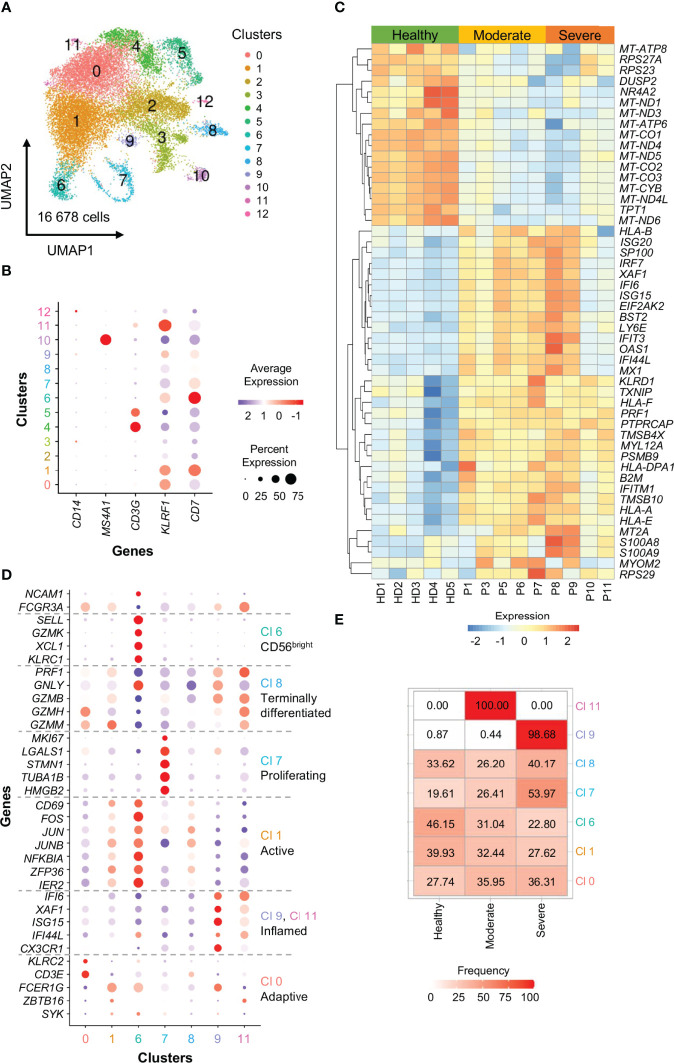
scRNA-seq profiling of NK cells from COVID-19 patients.**(A)** A total of 16 678 cells were embedded by Uniform Manifold Approximation and Projection (UMAP) plots in 13 clusters at a resolution level of 0.2. Each dot within the UMAP corresponds to one single cell colored according to cell cluster.**(B)** Ballon plots showing the expression of canonical NK cell markers in the 13 clusters identified as NK cells. Balloon size corresponds to the frequency of marker-positive cells and balloon color corresponds to the marker expression level of marker-positive cells.**(C)** Heatmap depicting the top 50 unique DEGs with adj. P value ≤ 0.05. Scale represents normalized counts centered and scaled across cells.**(D)** Ballon plots showing the expression of 33 NK cell markers to define cluster (Cl) identities. Balloon size corresponds to the frequency of marker-positive cells and balloon color corresponds to the marker expression level of marker-positive cells.**(E)** Heatmap showing the distribution of the 7 NK cell clusters among the 3 groups of subjects analyzed.

To further confirm the data present in literature, we analyzed scRNA-seq data from a publicly available dataset (67), by characterizing in detail the circulating NK cell compartment in 5 healthy donors, 5 moderate and 4 severe COVID-19 patients. Briefly, raw reads were processed using the Cell Ranger Single-Cell Software Suite (version 3.0.2; 10X Genomics) and aligned against the GRCh38 human reference genome. For quality check and downstream clustering analysis the Seurat pipeline was used (version 3.1.1; R version 3.6.1) (68). Each individual data set was processed separately and then integrated. By using SingleR, NK cell clusters were identified and re-clustered (**Figure 2A**). According to the expression of lineage markers, we excluded from the analysis: clusters 2 and 3 expressing low levels of KLRF1 and CD7, clusters 4 and 5 expressing the T-cell marker CD3G, cluster 10 expression the B-cell marker MS4A1 and cluster 12 expressing the monocyte marker CD14 (**Figure 2B**). In the 7 selected NK cell clusters we next studied the differentially expressed genes (DEGs) between moderate or severe COVID-19 patients and healthy individuals the expression of 33 NK cell markers to define cluster identities and their distribution among the 3 groups of subjects analyzed. **(** **Figures 2C–E****)**.

The data obtained showed that proliferating NK cells (cluster 7) is increased in severe patients. Moreover, in agreement with previous findings, our data demonstrated that inflamed CD56^dim^ NK cells expressing *CX3CR1* (cluster 9), expand in severely infected individuals (**Figure 2**), probably because these cells are not recalled to the lung given the reduced levels of CX3CL1, the ligand of CX3CR1, in BALF from COVID-19 patients (36, 69).

Circulating NK cells in SARS-CoV-2 infected subjects also show an effector phenotype characterized by an increased expression of cytotoxic molecules *PRF1* (Perforin) and *GZMB* (Granzyme B) at transcriptional level (**Figure 2C**) (36, 39).

Although their expression appears to be independent from disease severity, it has been observed that the expression of these cytotoxic granules on CD56^bright^ NK cells directly correlates with: IL-6 circulating levels, sequential organ failure assessment score, decreased PaO_2_/FiO_2_ ratio, and a general activation and upregulation of effector molecules within all NK cells. Furthermore, this phenotype is inversely correlated with the expression of the T cell immunoreceptor with immunoglobulin and ITIM domain (TIGIT) inhibitory checkpoint molecule, thus suggesting that the disease status and circulating cytokines could directly influence NK cell phenotype (36).

The activated and effector status of NK cells has been also confirmed in BALF as *GZMB*, *GZMA*, *PRF1*, *HAVCR2* (Tim-3), and *CCL4* are upregulated in COVID-19 patients compared to controls (36).

This activated pattern is typical of IFN-controlled cell activation programs and suggests an inflamed phenotype. In agreement, like others, we also observed an enrichment in type I IFN-related genes, including *ISG20, IRF7, XAF1, IFI6, ISG15, IFIT3, IFI44L, MX1, TXNIP* and *IFITM1* in NK cells from COVID-19 patients (**Figures 1**, **2C**). These data are also in line with the increased circulating levels of virus-induced type I and type II interferons (39, 66). The inflammatory phenotype of COVID-19 NK cells is particularly relevant in severe patients and especially during the first week after the disease onset (**Figures 2D–E**) (39, 66). In agreement, COVID-19 severe patients show increased plasma concentration of IFN-α, IFN-γ, IL-6 and TNF-α early after the disease onset (66). In this regard, our analyses demonstrated that NK cells from severe patients examined during the first week from the disease onset (P8 and P9) show a more inflammatory phenotype, characterized by higher gene expression of *ISG20, IRF7, XAF1, IFI6, ISG15, IFIT3, IFI44L, MX1* with respect to NK cells from severe patients analyzed at about 15 days after the disease onset (P10 and P11), as well as from moderate patients (P1, P3, P5, P6) (**Figure 2C**) (67).

The proinflammatory phenotype of NK cells in SARS-CoV-2 infection is further supported by several findings suggesting that CD16 is downregulated in COVID-19 patients during the acute phase as well as in convalescent subjects (**Figure 1**) (53, 70). Indeed, it has been reported that the downregulation of CD16 occurs in CD56^dim^ NK cells after their activation by target cells or after cross-linking of CD16 with antibodies, and results in an increased IFN-γ production (71, 72). Leem and coworkers, characterizing these CD56^dim^CD16^neg^ NK cells, demonstrated that they expand in the early phases of SARS-CoV-2 infection, they then rapidly decrease in mild patients while in severe patients the expansion of this subset lasts longer (73).

Moreover, the deregulation of genes involved in cellular metabolism and oxidative phosphorylation, including mitochondrial genes, further highlights the profound changes in cellular activation in the context of SARS-CoV-2 infection (**Figure 2C**) (39).

### 2.3 Impact of SARS-CoV-2 Infection on NK Cell Effector Functions

These activated and inflamed patterns suggest the involvement of NK cells in the early acute phase of SARS-CoV-2 infection and in COVID-19 pathogenesis.

Wheatear NK cells are able to detect SARS-CoV-2-infected cells remains largely unknown. Novel findings reported the direct interaction between NK cells and SARS-CoV-2-infected cells. Specific SARS-CoV-2 S protein peptides are capable of binding to the NKG2D receptor and increase NK cell cytotoxicity and IFN-γ production toward lung cancer cells (74). In addition, the non-structural protein 13 (Nsp13) of SARS-CoV-2 encodes for a peptide that forms stable complexes with HLA-E and prevents its binding to the inhibitory receptor NKG2A, thereby rendering target cells susceptible to NK cell attack. In line with these observations, NKG2A-expressing NK cells, that are mainly lung-resident (75), are particularly activated in patients with COVID-19 and proficiently limit SARS-CoV-2 replication in infected lung epithelial cells *in vitro* (52).

Nevertheless, several findings indicate that NK cells in SARS-CoV-2 infection can exhibit an exhausted phenotype. Indeed, programmed cell death protein 1 (PD-1), Lymphocyte-Activation Gene 3 (LAG-3), and TIGIT expression is higher in COVID-19 patients compared to healthy controls, while DNAM-1 and NKG2D-expressing NK cells are decreased in frequency (**Figure 1**) (54, 66, 70). The lower expression of NKG2D is also maintained in convalescent patients with asymptomatic and moderate history (53). Of note, the reduced NK cell expression of DNAM-1, together with the coinhibitory receptor TIGIT, identifies patients with a slow viral clearance (30).

Furthermore, it has been proposed that the increased circulating levels of IL-6 could contribute to lowering NKG2D expression (76).

In this regard, the local and systemic inflammation could also determine an impairment in circulating NK cell effector functions (77, 78).

In agreement, recent *in vitro* experimental findings showed a marked dysfunction of blood NK cells from COVID-19 patients, in particular those from severe ones, in terms of IFN-γ and TNF production, degranulation and killing ability against K562 target cells, as well as in the ability to control virus replication (**Figure 1**) (39, 66). Given the high expression of pro-inflammatory and immune-suppressive cytokines especially in severe patients, it is plausible that they could participate in determining the functional impairment of circulating NK cells in COVID-19 patients. As a proof of concept, plasma from severe COVID-19 patients resulted in a marked functional impairment of NK cells from healthy controls (66). New findings also demonstrated that Transforming growth factor beta (TGF-β) could play a main role in determining NK cell impairment in COVID-19 patients. Indeed, the early peak of TGF-β in hospitalized SARS-CoV-2-infected subjects is closely correlated with defective NK cell effector functions (**Figure 1**). Moreover, experimental evidence demonstrated that the *in vitro* administration of TGF-β or of serum from severe COVID-19 patients inhibits the ability of NK cells from healthy subjects to control SARS-CoV-2 replication, cell-mediated cytotoxicity and to perform cytotoxic responses and cytokine release. Furthermore, the presence of TGF-β-blocking antibodies, but not of neutralizing antibodies against IL-6, IL-10 or IL-15, can restore the NK cell effector functions (39). Despite these findings, the real ability of NK cells to lyse viral infected cells within the lung is still unknown. However, NK cells certainly contribute to determining lung pathology in COVID-19 patients. Indeed, both circulating and pulmonary COVID-19 NK cells expressed high levels of AREG (encoding for amphiregulin), an epidermal growth factor receptor ligand involved in pulmonary fibrosis. Furthermore, human lung fibroblasts co-cultured with NK cells from COVID-19 severe patients expressed high levels of the pro-fibrotic genes COL1A1 and ACTA2 and have a reduced frequency of active Caspase-3 with respect to NK cells from controls as well as from mild patients (66).

## 3 NK Cell Crosstalk With Other Immune Cells in SARS-CoV-2 Infection

Complex bidirectional interactions between NK cells and a variety of other immune cells are needed to support effective and long-lasting antiviral immune responses and to finely regulate the ability of NK cells to prevent excessive systemic inflammation during viral infections (79). Though these interactions can be crucially relevant to the clinical outcome of SARS-CoV-2 infection, they have been poorly investigated, so far.

### 3.1 NK Cell Crosstalk With Monocytes/Macrophages

NK cell crosstalk with monocytes/macrophages is mediated by cell-to-cell contact and soluble mediators that reciprocally potentiate cell recruitment and activation at the site of inflammation (80). Upon viral infection, NK cells secrete chemokines and cytokines, including macrophage inflammatory protein (MIP)1α, which recruits monocytes to the infected tissue and promotes their activation (79). In turn, activated macrophages release chemokines, including CXCL9, CXCL10, and CXCL11, which further recruit NK cells, and a wide range of pro-inflammatory and inhibitory cytokines, that finely tune the activation of NK cells (80). Evidence provided in COVID-19 patients suggests that the interaction with monocytes might impair NK cell recognition and killing of SARS-CoV-2-infected cells (81). Indeed, similarly to SARS-CoV-infected epithelial cells, inflammatory monocytes and macrophages release high amounts of IL-6 and TNF-α within infected tissues. In turn, both IL-6 and TNF-α can impair NK cell cytolytic functions: IL-6 through the IL-6/JAK/STAT3 signaling axis, with hyperactivated STAT3 exerting negative regulatory effects on NK cells (81, 82), while TNF-α by downregulating the expression of the natural cytotoxic receptor NKp46 (83) and upregulating the expression of the immune checkpoint Tim-3 on NK cell surface (84). Additional monocyte-related mechanisms contributing to NK cell dysfunction may be represented by a reduced secretion of IL-12 and IL-15, two cytokines that sustain NK cell activity and that are markedly reduced in the serum of severe COVID-19 patients (85). Notably, beyond their cytolytic activity directed against infected cells, NK cells play a very important role in the control of tissue homeostasis, by exerting negative feedback mechanisms on macrophages, aimed at preventing excessive inflammation in response to infections. Activated macrophages upregulate the expression of stress-inducible ligands, triggering NK cells (through the engagement of NKG2D receptor) to kill them and hampering the resolution of inflammation by a contra-regulatory immune mechanism (86). In SARS-CoV-2 infection, it has been hypothesized that the reduced cytotoxic activity of NK cells may also impair their homeostatic role and may therefore contribute to the hyperinflammation typically occurring in severe COVID-19 patients (76, 85).

This possibility may be supported by several mechanisms. First, TGF-β, which is increased in COVID-19 patients, inhibits NK cell cytotoxic activity by downregulating the expression of NKG2D, used by NK cells for exerting their homeostatic function (87, 88). Second, elevated IL-6, as observed in SARS-CoV-2-infected patients, has also the capacity to reduce the expression of NKG2D (89). Third, SARS-CoV-2 infection down-regulates activating NK cell ligands including MICA (90), and genetic variants that lead to lower cell surface expression of MICA and MICB are associated with more severe COVID-19 (91).

### 3.2 NK Cell Crosstalk With DCs

NK cells also interact with DCs, through cell-to-cell contact and soluble mechanisms, recently reviewed elsewhere (13). As for NK cell-monocyte crosstalk, also in the case of NK cells and DCs exists a bidirectional interaction, responsible on one hand for reciprocal cell activation, on the other hand for homeostatic control aimed at preventing excessive immune activation. Homeostatic control is achieved through DC killing by NK cells, this action being finely regulated by NK cell/DC ratios and by the interaction between DNAM-1 on NK cells and their ligands CD155 and CD112 on fully activated DCs (79, 92, 93). Reciprocal NK cell-DC activation is complicated by the heterogeneity of DCs that are composed of different subsets each endowed with functional specialization. Accordingly, the interaction of NK cells with different DC subsets may differentially affect adaptive immune responses. For instance, NK cells exposed to IL-2 or IL-12 can induce the maturation of type-1 conventional DCs, which in turn sustain type 1 immune responses through the development of T helper 1 and cytotoxic T cells, whereas NK cells exposed to IL-4 might favor tolerogenic or type 2 adaptive immune responses (94–96). At present, little is known about NK cell-DC crosstalk in COVID-19 patients. A recent study, a gene expression profile of peripheral blood mononuclear cells in SARS-CoV-2 infected patients at single-cell level, indicated that patients with severe disease have reduced pathways associated with NK cell-DC crosstalk, suggesting that dysregulation of immune crosstalk could be associated with COVID-19 severity (37). Moreover, the observation that in SARS-CoV-2 infected individuals the count of circulating NK cells is directly correlated with the level of anti-SARS-CoV-2 IgG antibodies suggests that NK cell-DC crosstalk in COVID-19 patients may also affect humoral adaptive immune responses, likely by indirectly promoting the secretion of IL-21 by T cells (97, 98).

### 3.3 NK Cell Crosstalk With Neutrophils

Also in the case of NK cell-neutrophil crosstalk, bidirectional interactions between NK cells and neutrophils have been demonstrated to control reciprocal cell activation, and protect from excessive immune activation (79). As for the crosstalk between NK cells and other immune cell types, interactions between NK cells and neutrophils rely on cell-to-cell contact and soluble mediators. Neutrophils can recruit NK cells to the infected tissue by secreting a wide range of chemokines, including CXCL9, CXCL10, and CXCL11, and can modulate NK cell survival, proliferation, cytotoxic activity and IFN-γ production *via* the generation of reactive oxygen intermediates, prostaglandins, and the release of granule components (99–101). Activated NK cells in turn can mediate the activation of neutrophils through the release of inflammatory cytokines and contact-dependent mechanisms (100). Notably, in order to counteract the accumulation of pathogenic neutrophils and the related detrimental consequences for the host, NK cells can kill neutrophils *via* NKp46 and Fas-dependent mechanisms (102). To the same aim, NK cell-derived IFN-γ directly inhibits neutrophil recruitment and survival (103). In COVID-19 patients, it has been hypothesized that the high levels of IL-6, IL-8 and IL-10 released by multiple cell types at the site of SARS-CoV-2 infection may alter the number and function of NK cells and neutrophils, thus compromising their mutual equilibrium (104). In facts, IL-8 and IL-6 are known to recruit and activate neutrophils, but they can also impair NK cell function *via* STAT3-dependent mechanisms (105). Furthermore, high levels of IL-6 and IL-10 have also been demonstrated to upregulate NKG2A expression on NK cells with a subsequent increment of its inhibitory action, thus compromising the balance between NK cells and neutrophils (104, 106). It has also been proposed that, in the lung microenvironment of COVID-19 patients, NK cells may interact with immature neutrophils and myeloid-derived suppressor cells, but details of these crosstalks still remain elusive (107).

## 4 NK Cell-Based Therapies in SARS-CoV-2 Infection

Given the crucial role of NK cells in antiviral immunity in general and their specific role in the immunopathogenesis of COVID-19, NK cell-based therapeutic approaches have been developed.

Herein, we will summarize the possible NK cell-based therapies against SARS-CoV-2 infection and the ongoing clinical trials updated at the time of submission of the review. Due to the continuously evolving landscape of clinical trials for COVID-19, the reader should be aware that in the meanwhile some information may have changed.

A first approach is represented by the administration of immunostimulants aimed at improving the *in vivo* NK cell activity in COVID-19 patients. In this regard, several bioactive molecules (i.e. IFN-α, IL-2, IL-12, and IL-15) have been used for the treatment of disorders characterized by impaired NK cell function (108). In particular, the administration of IL-12 and IL-15 can compensate for the NK cell dysfunction determined by the reduced secretion of these cytokines by monocytes (85).

In addition, as previously described, IL-2 and IL-12 secreted by NK cells play an important role in the context of NK cell-DC crosstalk, by promoting the maturation of type-1 conventional DCs, which in turn sustain type 1 immune responses (94–96). For these reasons, therapeutic approaches that aim at restoring a proper balance in the levels of these cytokines may represent a useful tool to improve the innate immune cell functionality and therefore to better sustain the adaptive immune responses.

Among these cytokines, IL-2 and IL-15 are the most used in clinical trials since they are involved in the processes of NK cell expansion and maturation (79, 109–112). In the context of COVID-19, a phase 2 clinical trial aimed at evaluating the efficacy of the daily administration of low-doses of IL-2 for 10 days in improving the clinical course and oxygenation parameters in patients with SARS-CoV-2-related ARDS was recently completed (NCT04357444). However, to date, the results emerging from the clinical trial are not available.

Nevertheless, the proinflammatory nature of certain cytokines, including IL-2 and IL-15, must be taken into account in the development of cytokine-based therapeutic approaches. In this context, elevated levels of IL-15 have been reported in association with chronic pulmonary inflammatory diseases and MERS-CoV infection (79). In addition, Sahoo and colleagues, by using an artificial intelligence-guided big data approach, showed the relevance of NK cell senescence induced by IL-­15/IL­15RA pathway in the development of severe or fatal COVID­-19 (113). In agreement, Liu and colleagues demonstrated that IL-15 plays a role in NK cell dysfunction observed in most severe COVID-19 patients (114). Therefore, although cytokine-based therapeutic strategies are less expensive and less time consuming than cell-based therapies, their use in clinics should be fine-tuned to avoid the further exacerbation of the inflammation in COVID-19 patients (107).

Considering that IL-6 can impair NK cell functions and that elevated level of IL-6 is a key feature of severe SARS-CoV-2 infection (51), clinical trials aiming to disclose the efficacy of drugs inhibiting IL-6 signaling are ongoing [review in (115)] and promising results deriving from the use of Tocilizumab, a humanized monoclonal antibody against IL-6 receptor, support the hypothesis that IL-6 axis represents a possible therapeutic target to treat severe COVID-19 patients by promoting NK cell functionality (33, 116).

A second approach of NK cell-based therapies is represented by the possible application of drugs that block NK cell inhibitory receptors, such as NKG2A. Since it has been reported that NKG2A is highly expressed by NK cells in COVID-19 patients and its expression has been associated with NK cell functional exhaustion, targeting NKG2A may improve NK cell immune responses (36, 51, 53).

A third and last approach is represented by therapeutic adoptive NK cells therapies (**Table 1**) (79).

**Table 1:** List of clinical trials proposed for COVID-19 treatment and based on primary and “off-the-shelf” NK cells.

NCT number	Title	Status	Study description	Study type
NCT04324996	Phase I/II Study of Universal Off-the-shelf NKG2D-ACE2 CAR-NK Cells for Therapy of COVID-19	Recruiting	Intervention: Biological (NK cells, IL15-NK cells, NKG2D CAR-NK cells, ACE2 CAR-NK cells, NKG2D-ACE2 CAR-NK cells)	Interventional; Phase 1/2
NCT04900454	Allogeneic Natural Killer (NK) Cell Therapy in Subjects Hospitalized for COVID-19	Recruiting	Intervention: Biological (DVX201)	Interventional; Phase 1
NCT04634370	A Phase I Clinical Trial on NK Cells for COVID-19	Not yet recruiting	Intervention: Biological (NK Cell infusion)	Interventional; Phase 1
NCT04280224	NK Cells Treatment for COVID-19	Recruiting	Intervention: Biological (NK Cells)	Interventional; Phase 1
NCT04365101	Natural Killer Cell (CYNK-001) Infusions in Adults With COVID-19	Active, not recruiting	Intervention: Biological (CYNK-001)	Interventional; Phase 1/2
NCT04578210	Safety Infusion of Natural Killer cells or Memory T Cells as Adoptive Therapy in COVID-19 pneumonia or Lymphopenia	Recruiting	Intervention: Biological (T memory cells and NK cells)	Interventional; Phase 1/2
ChiCTR2000031735	Clinical study for natural killer (NK) cells from umbilical cord blood in the treatment of novel coronavirus pneumonia (COVID-19)	Not yet recruiting	Intervention: Biological (NK cells)	Interventional; Phase 0
ChiCTR2000030944	Clinical study of human NK cells and MSCs transplantation for severe novel coronavirus pneumonia (COVID-19)	Not yet recruiting	Intervention: Biological (NK cells and MSC transplantation)	Interventional; Phase 1
IRCT20200417047113N1	Evaluating the safety and efficacy of allogeneic NK cells on COVID-19 induced pneumonia, double blind, randomized clinical trial	Recruitment complete	Intervention: Biological (NK Cells)	Interventional; Phase 1/2

NK cells used for therapeutic purpose can be obtained starting from granulocyte-colony stimulating factor (G-CSF)- mobilized peripheral blood mononuclear cells (G-PBMCs) or stem cells (79, 117, 118), by optimizing the culture condition to shift *in vitro* NK cell production to the highly cytolytic CD56^dim^ population in order to avoid the exacerbation of patient conditions consequent to the administration of the cytokine producer CD56^bright^ NK cells (79).

An additional NK cell-based therapeutic approach in the treatment of SARS-CoV-2 infection is represented by the infusion of adaptive/memory-like NK cells, endowed with higher functionality after appropriate activation with pro-inflammatory cytokines (53, 108). Data reported in literature support the hypothesis that adaptive NK cells, such as NKG2C^pos^CD57^pos^ NK cells, may be generated also in response to SARS-CoV-2 infection (36, 54, 55). Thus, their presence should be taken into account for the selection of convalescent donors in clinical trials for NK cell therapies (53). In this regard, Herrera and colleagues reported that the procedure of cell purification performed using a CliniMACS Plus cell separation system (Miltenyi Biotec) activates NK cells, making the NKG2C^pos^CD57^pos^ NK cell population more noticeable, as well as increasing the cytotoxic CD16^pos^ population. This could offer an advantage when transfusing this product to COVID-19 patients (53). Moreover, the use of plasmalyte with 40% AB serum and 10% DMSO ensured good results in terms of NK cell viability and functionality, allowing “off-the-shelf” NK treatments (53).

Therapeutic NK cells can be obtained also from immortalized human NK cell lines which are genetically engineered (79, 119). They represent a suitable choice for COVID-19 patients since they produce low levels of interferon (34). The technology of chimeric antigen receptor (CAR)-NK cells, successfully applied in oncology, allows to design NK cell lines that specifically express receptor(s) of interest thus increasing the ability of NK cells to recognize specific antigens and to thus eliminate specific targets. In the context of COVID-19 treatment, the used of different lines such as NKG2D-ACE2 CAR-NK cells and ACE2 CAR-NK cells in combination with an IL-15 superagonist and Granulocyte-macrophage colony-stimulating factor (GM-CSF) neutralizing single-chain variable fragments are currently under investigation (NCT04324996) (79, 120). By targeting the S protein of SARS-CoV-2 and NKG2DL on the surface of infected cells with ACE2 and NKG2D, respectively, these therapeutic strategies aim at identifying SARS-CoV-2 particles and SARS-CoV-2 infected cells for their effective removal.

## 5 Conclusions

Currently available data in literature demonstrate that COVID-19 severity depends on two elements that mutually affect: the efficacy of anti-SARS-CoV-2 NK activity on one side and the effects of SARS-CoV-2 on NK cell functionality on the other one. On this basis, therapeutic approaches aimed at “fine tuning” NK cells activity in the context of SARS-CoV-2 infections have been proposed, to balance their beneficial antiviral and their detrimental pathologic action in COVID-19 patients.

To deepen the knowledge on the role of innate immunity in SARS-CoV-2 infection, further studies should be performed to investigate the crosstalk between NK cells and the other innate immune cell populations during the acute phase of the infection. This information is essential to disclose the role of this crosstalk in COVID-19 pathogenesis. Indeed, a deeper comprehension of the NK cell crosstalk with other immune cells will allow to better understand how innate immune cells can modulate the adaptive immune responses and could also allow the identification of novel predictors of clinical outcome.

Up to now, the current knowledge regarding NK cells and SARS-CoV-2 relies on studies focused on the acute phase of the infection and on studies comparing COVID-19 patients stratified based on disease severity. Future studies on subjects that are convalescent after SARS-CoV-2 infection are required to assess the persistence of the NK cell impairment observed in acute phase of the infection and to investigate the long-term impact of natural infection in inducing the development of NK cells with adaptive-like properties that could guarantee protection from re-infection. This aspect is of particular interest in the context of Long-COVID. Indeed, a deeper characterization of NK cells in patients experiencing COVID-19 sequelae could allow a better comprehension of the molecular mechanisms driving NK cell impairment in COVID-19 and could also allow the development of effective NK cell-based therapeutic approaches to treat Long-COVID patients.

Finally, the role of NK cells in determining a long-term anti-SARS-CoV-2 protection also after vaccination is another aspect that deserves to be investigated more in detail.

In this context, very recent evidence suggests that the frequency of NKG2C^pos^ NK cells before the vaccination can positively influence the anti-SARS-CoV-2 antibody titers following two doses of BNT162b2 mRNA vaccine (121). However, by comparing the NK cell responses in subjects receiving two doses of inactivated SARS-CoV-2 vaccine (CoronaVac) and who develop or not COVID-19 after vaccination or subjects experienced for SARS-CoV-2 infection and infused or not with CoronaVac, any differences in terms of IFN- γ release upon overnight stimulation with aspecific NK cell activation stimuli have been observed (122).

An extensive phenotypic and functional characterization of NK cells by using SARS-CoV-2 specific stimuli in vaccinated subjects will allow to assess whether anti-SARS-CoV-2 vaccines could stimulate the development of NK cells with higher effector functions or adaptive-like properties that could guarantee a faster and more efficient response in preventing SARS-CoV-2 infections and/or severe COVID-19 forms.

## Author Contributions

CDV, FC, AV, SDB, JM, and DM wrote the manuscript and critically reviewed the manuscript. ST and NC analyzed publicly available scRNAseq data. CDV, FC, ST, and NC draw the figures. All authors gave the final approval to the manuscript.

## Funding

This work was supported by Italian Ministry of Health “Bando Covid-19” (COVID-2020-12371640 to DM) and by Fondazione Cariplo-Fondazione Umberto Veronesi (Proposal 2020-1376 to DM). ST is a recipient of competitive fellowship awarded from the Data Science in Medicine and Nutrition (DASMEN) Ph.D program from Humanitas University. We also thank the financial support from Fondazione Romeo ed Enrica Invernizzi.

## Conflict of Interest

The authors declare that the research was conducted in the absence of any commercial or financial relationships that could be construed as a potential conflict of interest.

## Publisher’s Note

All claims expressed in this article are solely those of the authors and do not necessarily represent those of their affiliated organizations, or those of the publisher, the editors and the reviewers. Any product that may be evaluated in this article, or claim that may be made by its manufacturer, is not guaranteed or endorsed by the publisher.
